# Comparison of Patient-Reported Quality of Life Following Direct-to-Implant Prepectoral and Subpectoral Breast Reconstruction Using BREAST-Q: A Randomized Controlled Trial

**DOI:** 10.1055/a-2407-9183

**Published:** 2024-11-13

**Authors:** Diana L Dyrberg, Farima Dalaei, Martin Sollie, Camilla Bille, Vibeke Koudahl, Jens A Sørensen, Jørn B Thomsen

**Affiliations:** 1 Department of Plastic Surgery, Odense Universitetshospital, Odense, Denmark

**Keywords:** breast reconstruction, quality of life, BREAST-Q, breast implant, patient-reported outcome

## Abstract

**Background** Direct-to-implant breast reconstruction (DIR) is becoming more and more accepted. There is a lack of high-quality studies assessing differences in patient-reported quality of life (QoL) between different implant placement methods. The aim of this randomized controlled (clinical) trial was to compare QoL between women reconstructed by sub- or prepectoral implant placement.

**Methods** We included women over 18 years eligible for DIR. Patients were randomly assigned to reconstruction by subpectoral or prepectoral implant placement. Assessment of QoL and patient satisfaction was made using the BREAST-Q questionnaire for postmastectomy breast reconstruction and compared between the sub- and prepectoral reconstructed groups preoperatively and after 3 and 12 months of follow-up.

**Results** A total of 42 women were allocated to sub- or prepectoral reconstruction with 21 patients in each group. There were no differences in patient characteristics between groups. Regarding all the selected BREAST-Q scales: (1) satisfaction with the reconstructed breast, (2) satisfaction with the breast implant, (3) satisfaction with the overall outcome, (4) psychosocial well-being, (5) sexual well-being, and (6) physical well-being—we found no significant differences between the two groups. Assessing each group independently we found, that in both groups sexual well-being improved after surgery postoperatively compared to the preoperative scores.

**Conclusion** We found high satisfaction and QoL following both sub- and prepectoral breast reconstruction. We found no significant differences between groups suggesting both methods for DIR can be used. Despite our high-quality data, a larger sample size and longer postoperative follow-up are needed to further investigate the differences in QoL between sub- and prepectoral breast reconstruction.

## Introduction

Mastectomy is a common treatment for breast cancer and is increasingly being used as a prophylactic measure in women at high risk of developing cancer. As a method of reconstruction, direct-to-implant breast reconstruction (DIR) is gaining acceptance. 1 DIR has proven to be time-saving with both physical and financial advantages compared to two-stage breast reconstructions. 2 3

When performing DIR, two main approaches are being used; the implant is either placed in a subpectoral pocket, under the pectoralis major muscle (PMM), or in a prepectoral pocket leaving the PMM undisturbed. Regardless of the pocket plane, a biological or synthetic mesh can be used to support the implant. In the subpectoral approach, one sheet of the mesh is used to create a “hammock” to support the inferior and lateral parts of the implant. This method was first described two decades ago. 4 When the prepectoral approach is used, the most common technique is to create a full hammock of mesh that covers the implant. This technique was first described in 2015 and since then the benefits and drawbacks of the different pocket planes have been described. 5

Since the introduction of acellular dermal matrices in 2005, the dual-plane submuscular pocket has been increasingly used worldwide. 6 This technique has proven to be a safe and effective way of reconstructing the breast, the main benefits being a vascularized cover protecting the implant, reduced rippling and implant visibility, as well as a lower incidence of capsular contracture. 7 8 Some of the disadvantages are related to muscle coverage which may cause breast animation deformity (BAD). BAD is an unwanted movement of the PMM caused by a compressing action on the underlying implant resulting in a superolateral movement of the whole breast, breast skin, or nipple–areolar complex. 9 Besides being a cosmetic problem, some experience pain and discomfort in relation to BAD. 10 After subpectoral breast reconstruction, it has been shown that all women experience some degree of BAD. 9 10

Recently there has been an increased focus on prepectoral breast reconstructions, 11 and it has been shown that the two pocket planes are comparable regarding postoperative complication rates. 12 When leaving the PMM undisturbed, patients report less postsurgical pain, and the degree of BAD can be reduced or even avoided. 13 For the reconstructed women, the main drawback of placing the implant prepectoral with a subcutaneous tissue cover only is related to insufficient thickness of the postmastectomy skin flaps. This seems to cause an increased risk of rippling and visibility of the implants. 13

When comparing sub- and prepectoral DIR focus has mainly been on assessing complications, cosmetic outcomes, and avoidance of BAD. The final choice of pocket plane is often based on the patient's preference along with the surgeon's experience. However, the main reason for DIR is to restore a naturally appearing breast and thereby increase patients' health-related quality of life (HRQOL). When evaluating breast reconstruction regarding different pocket planes, we must therefore keep the patient's perception in mind.

In health care, there has been a general shift towards patient-centered care. Patient-reported outcome measures (PROMs) have gained more attention. 14 BREAST-Q is a reliable, tested, and psychometric-validated PROM developed to assess HRQOL and patient satisfaction in women undergoing mastectomy, breast-conserving therapy, and breast reconstruction. 15 16 Since the introduction of BREAST-Q, several studies have demonstrated improved HRQOL in breast-reconstructed women compared to mastectomy alone. 17 Still, there is a lack of high-quality studies evaluating HRQOL between different implant placement methods. Going through the literature, most studies comparing sub- and prepectorally reconstructed women have used self-developed nonvalidated questionnaires. 18 19 Therefore, this randomized controlled study aimed to investigate the HRQOL of patients undergoing DIR using the BREAST-Q to compare patients reconstructed with subpectoral or prepectoral DIR.

## Methods

The data presented in this paper were collected as part of a randomized controlled (clinical) trial (RCT). The data presented in this are secondary outcomes and the power calculations were not based on the PROM data entailed in this paper, but were based on the risk of developing BAD in the two groups. The power calculations have been published in detail and described with the primary outcome measure, a comparison of the degree of BAD between the two groups. 20 This trial was primarily conducted to investigate differences in incidences and degrees of BAD following DIR with either subpectoral or prepectoral implant placement. A detailed description of the study design, data collection, and data management has previously been published by the authors. 21

We conducted an RCT in accordance with the Consolidated Standards of Reporting Trials guidelines. Patients were enrolled from two departments of Plastic Surgery in Denmark.

Between April 2017 and March 2020, all women over 18 years eligible for unilateral or bilateral DIR after a therapeutic or prophylactic skin- or nipple-sparing mastectomy, were assessed for eligibility. 20 21 Those meeting the criteria were invited to participate in the trial and informed consent was obtained prior to enrollment. Enrolled patients were evaluated at baseline including interview and clinical examination. Each patient was informed that the final screening for eligibility would be carried out during surgery, where the mastectomy flaps were assessed for thickness and viability, ensuring the tissue was suitable for prepectoral implant placement if randomized to this technique. When evaluated as suitable, patients were allocated to DIR with either sub- or prepectoral implant placement in a ratio of 1:1. The randomization was conducted by opening an envelope containing the allocation to one of the two intervention groups. There was no blinding of participants or investigators following surgery. The exclusion criteria were prior or planned radiation therapy to the breast, tobacco usage, hypertension treated with more than one drug, breast ptosis >2 measured by Regnault's ptosis scale, 22 body mass index (BMI) <22 or > 32 kg/m ^2^ , dementia, or psychiatric disorders making patients incapable of providing informed consent.

Breast reconstructions were performed in a standardized fashion as previously described by the authors. 23 For the subpectoral reconstruction the PMM insertion was divided inferomedially using monopolar cautery allowing partial muscle coverage of the implant. The inferior part of the implant was covered by an acellular mesodermal matrix (Meso BioMatrix® size 8 × 16 cm; AMM), which was sutured with vicryl 2.0 sutures to the edge of the muscle and the inframammary crease. Two pieces of AMM sutured together by vicryl 2.0 sutures were used for prepectoral reconstructions and sutured with vicryl 2.0 sutures circumferentially along the breast footprint to the PMM. The same postoperative regime was used for all patients.

To evaluate the HRQOL of patients undergoing DIR, we used the validated Danish version of the BREAST-Q. 22 At baseline evaluation in the outpatient clinic, the preoperative module of BREAST-Q was handed out directly. At 3- and 12-month follow-ups, the postoperative module of the BREAST-Q questionnaire for postmastectomy breast reconstruction (version 1.0) was administered. 16 We selected the following scales of the postoperative BREAST-Q: (1) satisfaction with breast, (2) satisfaction with breast implant, (3) satisfaction with outcome, and the quality of life (QoL) domains; (4) psychosocial well-being, (5) sexual well-being, and (6) physical well-being. We excluded scales regarding abdominal donor site, satisfaction with information, and satisfaction with the surgeon. For each scale, the raw scores were computed from the responses to the items, which were added together and converted to a Rasch-transformed scale (ranging from 0 to 100). 24 The item responses were collected in paper forms and later entered into the secure REDCap Database System by double data entry. 25 The trial was registered on clinicaltrials.gov (NCT03143335) and approved by The Danish Committee on Health Research Ethics (S-20160160) as well as the Danish Data Protection Agency (17/13640).

### Statistical Analysis

Descriptive statistics were used to summarize demographic data and type of implant, laterality of reconstruction, and mean volume of implants. Data are presented as means and standard deviations (SDs) or median and interquartile ranges. For all BREAST-Q scale items, raw scores were summed and converted to Rasch-transformed scores for each scale from 0 (worst) to 100 (best). We used the Qscore Software BREAST-Q Version 1.0 ( https://qportfolio.org/score-breast-q-breast-cancer-2/ ) for the preoperative and postoperative reconstruction modules. The Rasch-transformed scores for each scale were compared between the prepectoral and subpectoral implant placement group with an independent-sample *t* -test for normally distributed data or the Mann–Whitney U test for non-normally distributed data. All scores were reported as means and SDs (normal distribution) or medians and interquartile range (non-normal distribution). A *p* -value of 0.05 was deemed statistically significant. Preoperative scores from each group (prepectoral and subpectoral, respectively) were compared with 3- and 12-month postoperative scores of the same group with multiple comparison tests with Bonferroni adjustment. All statistical analyses were performed using SPSS, version 26 (IBM SPSS Statistics, Version 26.0. Armonk, NY: IBM Corp.), Qscore, and GraphPad Prism 7.0 (GraphPad Software Inc., CA).

## Results

Between April 2017 and March 2020, 69 women were assessed for eligibility. A total of 53 patients were randomized and allocated to sub- or prepectoral DIR, and finally, 21 women in the subpectoral group and 21 in the prepectoral group completed the trial and were eligible for analysis. A detailed description and flowchart of patient inclusion have been previously published. 20 The completion of this study was challenged by the coronavirus disease 2019 (COVID-19) pandemic as all our risk-reducing procedures were paused during the pandemic and the inclusion period was extended from 1 to 3 years accordingly.

Baseline characteristics after randomization were similar in both groups ( Table 1 ). In the subpectorally reconstructed group, the mean age was 50 ± 10.2 years and the mean BMI was 26.8 ± 2.25 kg/m ^2^ . For the prepectorally reconstructed group, the mean age was 49.4 ± 10.9 years and the mean BMI was 25.5 ± 2.4 kg/m ^2^ . In the subpectoral group, 28.6% had a bilateral reconstruction compared to 57.2% in the prepectoral group. The duration of surgery was similar between groups: 176 (121–354) minutes in the subpectoral group and 184 (130–333) minutes in the prepectoral group. The length of follow-up (days) was comparable between groups (399.4 vs. 390.4, *p* = 0.42).

**Table 1:** Demographics

Characteristics	Descriptive statistics		
	Subpectoral	Prepectoral	*p* -Value
Number of patients, *N*	21	21	–
Age, mean ± SD, years	50.0 ± 10.2	49.4 ± 10.9	0.84
BMI, mean ± SD, kg/m ^2^	26.8 ± 2.25	25.5 ± 2.4	0.08
Laterality of reconstruction, *N* (%)			
Unilateral	15 (71.4)	9 (42.8)	0.12
Bilateral	6 (28.6)	12 (57.2)	–
Duration of surgery, median (range)	176 (121–354)	184 (130–333)	0.56
Length of follow-up, mean ± SD	399.4 ± 36.7	390.4 ± 34.5	0.42

Abbreviations: BMI, body mass index; SD, standard deviation.

Data regarding complications, implants, and indications for surgery are described in a previous paper comparing the degree of BAD between groups. 20

All the preoperative and postoperative BREAST-Q questionnaires were completed by all patients (100%). When analyzing and comparing results between the sub- and prepectorally reconstructed groups, we found no significant differences in any of the domains investigated, neither in satisfaction nor in QoL ( Table 2 ). There were no significant differences in the mean preoperative scores for satisfaction with breasts between sub- and prepectorally reconstructed groups ( Table 2 ). The same applied to the postoperative scores regarding satisfaction with breast, satisfaction with implant, and satisfaction with outcome where no significant differences could be found ( Table 2 ).

**Table 2:** BREAST-Q scores

Preoperative (mean, SD)	Scale	Prepectoral ( *n* = 21)	Subpectoral ( *n* = 21)	Sig.
	Satisfaction with breast	68.71 (19.96)	70.05 (19.67)	ns
	Psychosocial well-being	79.76 (16.67)	72.43 (17.88)	ns
	Physical well-being	86.90 (11.31)	83.90 (09.76)	ns
	Sexual well-being	68.85 (20.63)	62.05 (15.92)	ns
Postoperative	Scale	Prepectoral ( *n* = 21)	Subpectoral ( *n* = 21)	Sig.
3 months postoperative	Satisfaction with breast	71.95 (18.51)	63.62 (15.87)	ns
	Satisfaction with breast implant	71.33 (21.83)	73.48 (19.84)	ns
	Satisfaction with outcome	80.86 (14.41)	75.95 (16.10)	ns
	Psychosocial well-being	66.65 (21.03)	60.24 (25.81)	ns
	Sexual well-being	82.14 (13.78)	74.67 (13.67)	ns
	Physical well-being	89.90 (20.81)	86.48 (18.80)	ns
12 months postoperative	Satisfaction with breast	72.81 (16.62)	67.90 (17.21)	ns
	Satisfaction with breast implant	77.67 (17.07)	73.57 (15.05)	ns
	Satisfaction with outcome	85.14 (13.19)	75.00 (19.40)	ns
	Psychosocial well-being	66.50 (13.89)	62.09 (20.12)	ns
	Sexual well-being	84.38 (15.63)	77.80 (16.46)	ns
	Physical well-being	89.90 (20.81)	87.09 (23.74)	ns

Abbreviations: ns, no statistically significant difference; SD, standard deviation; Sig., level of significance ( *p* ≤ 0.05).

BREAST-Q scores in the prepectoral and subpectoral groups: preoperatively, and 3 and 12 months postoperatively.

**Fig. 1:**
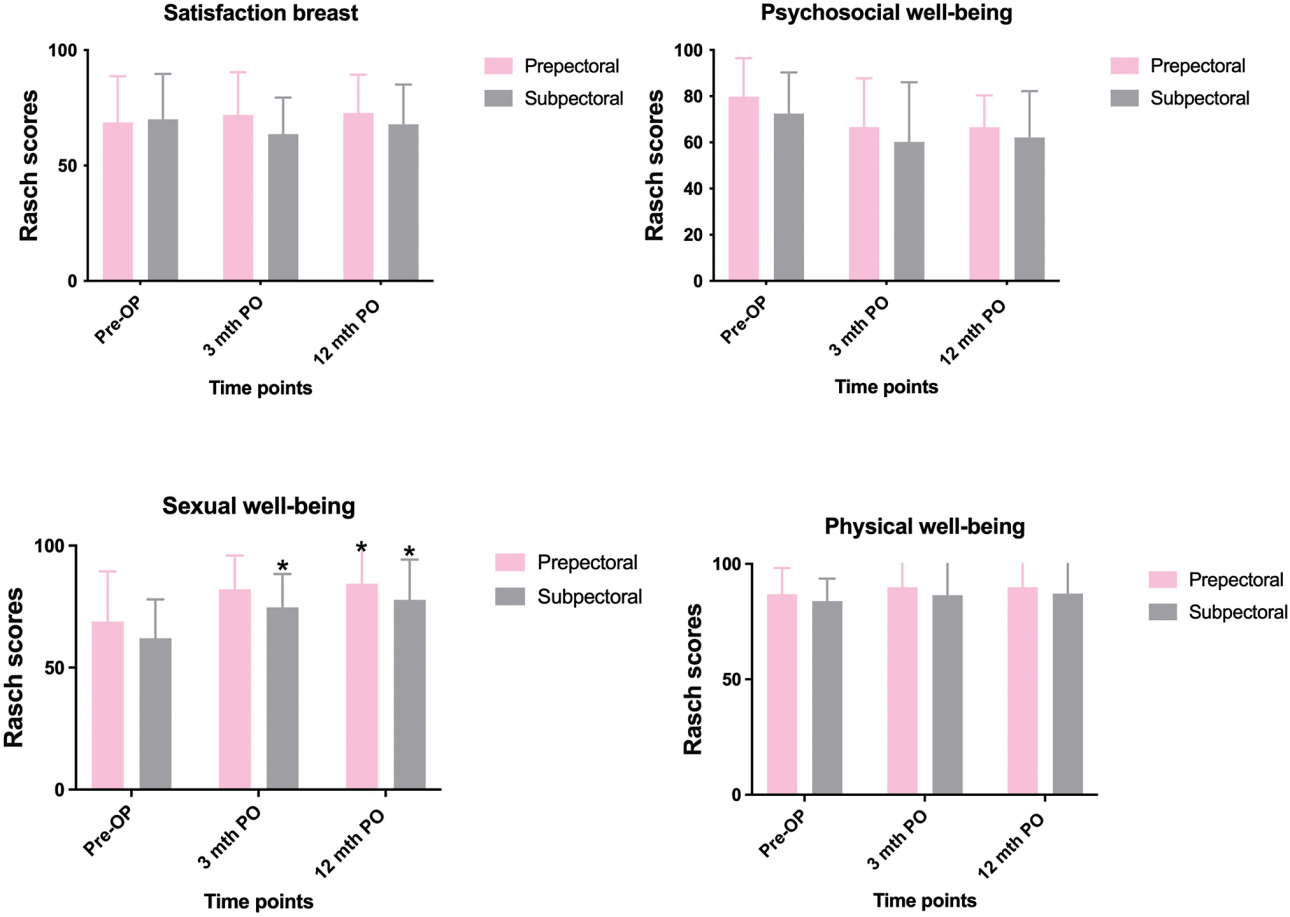
Mean change in BREAST-Q scores between groups. OP, operative; PO, postoperatively. * significant ( *p* < 0.05).

Correspondingly, when assessing HRQOL and comparing results between the sub- and prepectorally reconstructed groups, we found no significant differences in either physical, psychosocial, or sexual well-being ( Fig. 1 ). When assessing each group independently we found, that the prepectoral group scored significantly higher in sexual well-being 12 months postoperatively compared to the preoperative scores (84.38 vs. 68.85, Bonferroni-adjusted *p* -value [0.03]). The same tendency was seen when comparing the preoperative scores with the 3-month follow-up scores, however not significant (68.85 vs. 82.14), Bonferroni-adjusted *p* -value (0.07; Fig. 1 ). In the subpectoral group, patients had significantly higher sexual well-being scores at both 3- and 12 months postoperatively compared to preoperative scores (62.05 vs. 74.57), Bonferroni-adjusted *p* -value (0.03), and (62.05 vs. 77.8), Bonferroni-adjusted *p* -value (0.01), respectively. When assessing physical and psychosocial well-being in each group separately, no statistically significant differences could be found ( Fig. 1 ).

## Discussion

We achieved a 100% response rate and compared BREAST-Q scores assessed preoperatively, 3 and 12 months postoperatively from sub- and prepectorally DIR reconstructed women. Overall, we found no significant differences in BREAST-Q scores between the two groups, neither in patient satisfaction nor HRQOL after the reconstruction. Correspondingly, we could not detect any significant differences in assessing each group separately by comparing preoperative scores to 3- and 12-month postoperative scores, except in the HRQOL scale of sexual well-being. Interestingly, patients scored significantly higher in sexual well-being postoperatively compared to preoperative scores in both groups.

Mundy et al reported normative scores of the general population and compared the scores to previously published BREAST-Q scores. Our BREAST-Q *preoperative* scores were comparable to normative data published by Mundy et al, 17 except in the subtheme “physical well-being” where our scores were slightly below the norm (83.90–86.90 vs. 93). The marginally lower preoperative scores may be explained by pain following biopsy or sentinel lymph node dissection. Patients' physical well-being might also be affected by anxiety, the circumstances of going through surgery soon, and not knowing how everything will turn out in the end.

When comparing our *postoperative* scores to the same normative data by Mundy et al, patient satisfaction was above the normative scores regarding subthemes “satisfaction with breast” and “sexual well-being” and still slightly below in “physical well-being” (86.48–89.90 vs. 93). Postoperative lower scores in “physical well-being” compared to normative scores can be explained by multiple factors such as scar tissue, loss of feeling in the area, damage to the PMM, and overall discomfort. Furthermore, physical limitations after breast reconstruction could be a reason. Even though our findings in “physical well-being” were below the normative scores by Mundy et al, our data corresponded to the previously published BREAST-Q scores reported by the same authors that reported lower “physical well-being” scores after implant-based breast reconstruction (76 vs. 93). 17

If the low scores were solely related to discomfort and pain after reconstruction, one would expect scores to be higher at the 12-month follow-up. However, this was not the case as we found no significant differences between 3- and 12-month scores. Regarding our small sample size of 42 patients, a type 2 error is possible; however, we found our data on HRQOL between sub- and prepectoral groups comparable to findings by other studies. 26 27 28 29 30 Li et al conducted a systematic review and meta-analysis in 2019 comparing sub- and prepectorally reconstructed groups regarding complications, oncological safety, patient-reported outcomes, and pain. Corresponding to our findings, they found no significant differences between sub- and prepectorally reconstructed groups utilizing the BREAST-Q to evaluate PROM by assessing 16 comparative studies. 27 Several other studies report no difference in HRQOL between sub- and prepectoral groups; a prospective study by Baker et al aimed to compare differences in PROM between sub- and prepectorally reconstructed groups. They included 40 patients and 31 returned the BREAST-Q revealing no significant differences between groups. 28 Furthermore, a retrospective study by Walia et al aimed to compare HRQOL in patients reconstructed with tissue expanders. They identified 26 in the prepectoral group and 109 in the subpectoral group, which completed the BREAST-Q questionnaire and found no significant differences between groups. 29 Manrique et al also conducted a retrospective study evaluating BREAST-Q between 33 prepectorally reconstructed women and 42 subpectorally reconstructed women. After 20 months of follow-up, they found no significant differences in BREAST-Q scores. 26 Lastly, a retrospective study by Ng et al assessed HRQOL by BREAST-Q in 80 patients after DIR and found patients in the prepectorally reconstructed group reporting a significantly higher score in the “satisfaction with breast” subtheme (68.9 vs. 57.5; *p* = 0.036), but no significant differences in the other subthemes. 30 Correspondingly, we found no significant differences comparing sub- to prepectorally reconstructed groups in any of the applied BREAST-Q scales after reconstruction. There was a trend towards slightly higher satisfaction in the prepectoral group but no significant differences could be detected. Quite recently, a systematic review and meta-analysis has been published regarding QoL and pain related to reconstruction using either prepectoral and/or subpectoral implant placement. 31 In this study, patients reported higher BREAST-Q scores after prepectoral breast reconstruction.

Surprisingly, when analyzing each group separately, we found higher sexual well-being scores postoperatively compared to preoperative scores. The increased postoperative sexual well-being scores found in this study might be a result of several things. Patients might score low preoperatively when they are influenced by a recent cancer diagnosis that can induce a vulnerability prior to surgery, and sex may very well be the last thing on their minds. This vulnerability might disappear postoperatively as the patients have survived and fought cancer, a sense of relief. 32 We do not know if the patients were sexually active pre- or postoperatively in our population, nor do we know their marital status. However, our focus was to investigate differences between sub- and prepectorally reconstructed groups, and with that in mind, both groups were indifferent. More research is needed to investigate the change in sexual well-being and sexual function postoperatively in women undergoing DIR.

In a previously published retrospective study, we found a significant difference between the degree of BAD when comparing sub- and prepectorally reconstructed women. 9 With this in mind, and since BAD has previously been suggested to have a negative impact on HRQOL, 10 we would have expected the nuisance of BAD to be reflected in the HRQOL scores in our subpectorally reconstructed group. However, we found no significant difference in HRQOL reflecting the choice of pocket plane. Thus, patients may not be as affected by animation deformity as presumed by surgeons. A shift towards the prepectoral plane might not be the answer to all problems, even though it eliminates BAD. 33 Baker et al found no significant differences in HRQOL between pre- and subpectorally reconstructed groups corresponding to our findings. However, when analyzing outcomes from individual questions, they found significantly more patients in the prepectoral group either dissatisfied or very dissatisfied with the amount of rippling and visibility of the implant. 28 Implant visibility and rippling increase the need for correctional surgery, including fat grafting, which might negatively affect HRQOL with a risk of affecting all the advantages of the prepectoral plane. Nevertheless, careful evaluation of postmastectomy skin flaps is crucial when choosing the prepectoral plane.

In this RCT, it seems that both methods for DIR can be used with good satisfaction and HRQOL. The data presented here are, however, secondary endpoints, and therefore the power calculation is not performed for HRQOL as an outcome. The total number of 21 patients in each group could have insufficient power.

We did not examine if there was a difference in PROM between unilateral and bilateral cases, the sample size is too small for that; however, this is an important topic that could be interesting to look at in future and larger studies.

Conclusions drawn regarding pocket planes and their impact on satisfaction and HRQOL should therefore be made carefully. If any differences between the two DIR methods exist, they might be too insignificant to be detected in our small study sample (type 2 error). Furthermore, we cannot rule out that differences between groups would be revealed after a longer follow-up time, but we did, however, not find any significant differences between 3- and 12-month follow-up scores between the groups. This might suggest that HRQOL does not change over time. Furthermore, our findings were comparable to the previously mentioned meta-analysis by Li et al that correspondingly found no significant differences between sub- and prepectorally reconstructed groups utilizing the BREAST-Q. 27

This is an RCT where we used the BREAST-Q questionnaire, a reliable, tested, and psychometric-validated PROM. 16 Despite the limitations mentioned above, we conclude that women who undergo DIR are likely to report high satisfaction and HRQOL and have increased sexual well-being postoperatively. Nevertheless, HRQOL comparing implant-based reconstructions by different techniques is a subject that needs further investigation. RCTs with larger sample sizes and longer follow-ups are needed to accommodate deviations. We, as surgeons, influence the operative recommendations and choice of the surgical method. Still, in the end, the final and most important goal is to help our patients reach a high degree of satisfaction with their surgical outcomes.
